# Supplementary material for: A Humanized Yeast Phenomic Model of Deoxycytidine Kinase to Predict Genetic Buffering of Nucleoside Analog Cytotoxicity
Source: Genes (Basel). 2019 Sep 30;10(10):770. doi: 10.3390/genes10100770 (PMC6826991; doi:10.3390/genes10100770)
Supplement: Supplementary file 1 [file genes-10-00770-s001.zip › Additional_Files/Additional_File7_GOTermHeatmaps.zip!/Additional_File7_GOTermHeatmaps/Arp2_3_complex-mediated_actin_nucleation.pdf]

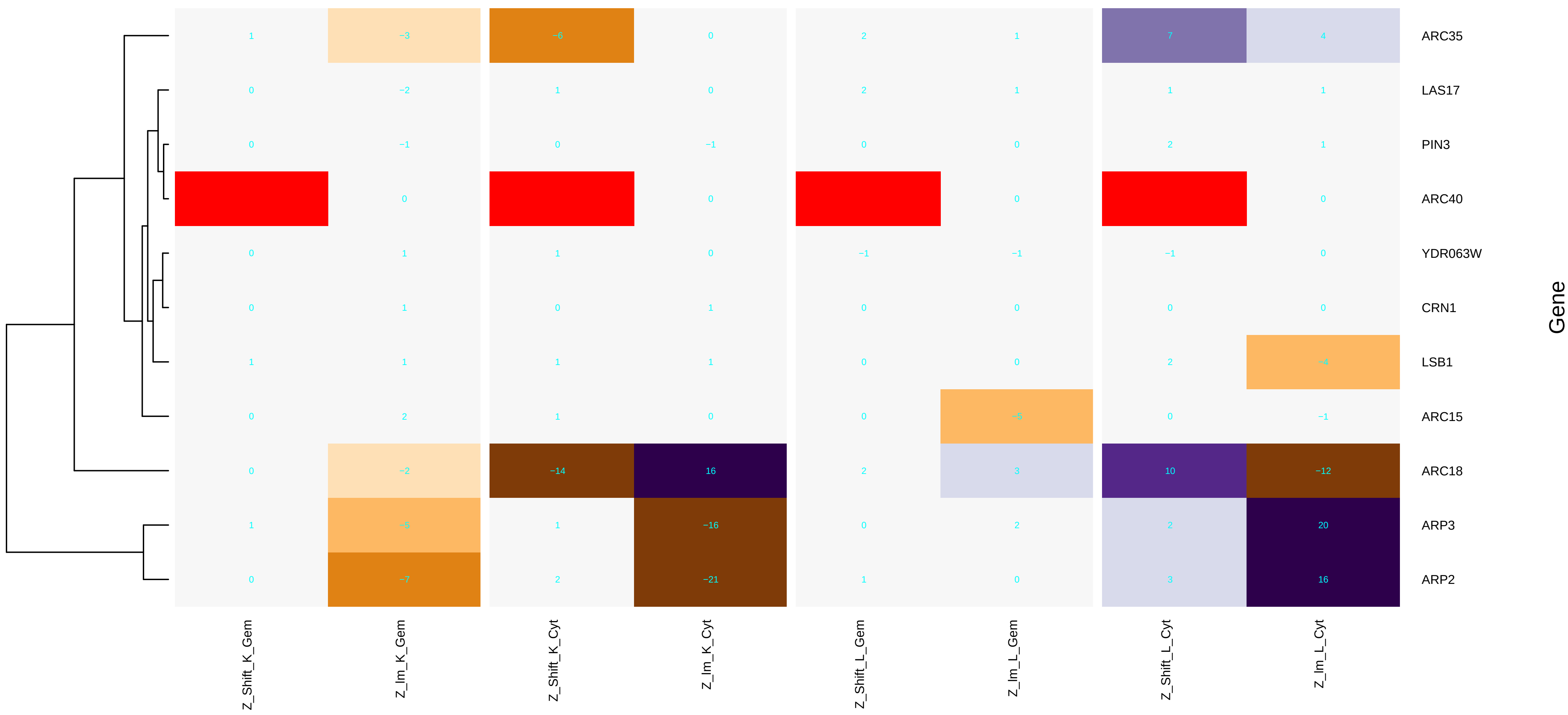

ARC35 1 −3 −6 0 2 1 7 4
LAS17 0 −2 1 0 2 1 1 1
PIN3 0 −1 0 −1 0 0 2 1
ARC40 0 0 0 0
YDR063W 0 1 1 0 −1 −1 −1 0
CRN1 0 1 0 1 0 0 0 0
LSB1 1 1 1 1 0 0 2 −4
ARC15 0 2 1 0 0 −5 0 −1
ARC18 0 −2 −14 16 2 3 10 −12
ARP3 1 −5 1 −16 0 2 2 20
ARP2 0 −7 2 −21 1 0 3 16
Z_Shift_K_Gem
Z_Im_K_Gem
Z_Shift_K_Cyt
Z_Im_K_Cyt
Z_Shift_L_Gem
Z_Im_L_Gem
Z_Shift_L_Cyt
Z_Im_L_Cyt
Gene
